# Nomograms for Predicting the Lymph Node Metastasis in Early Gastric Cancer by Gender: A Retrospective Multicentric Study

**DOI:** 10.3389/fonc.2021.616951

**Published:** 2021-09-29

**Authors:** Wannian Sui, Zhangming Chen, Chuanhong Li, Peifeng Chen, Kai Song, Zhijian Wei, Hu Liu, Jie Hu, Wenxiu Han

**Affiliations:** ^1^ Department of Gastrointestinal Surgery, The First Affiliated Hospital of Anhui Medical University, Hefei, China; ^2^ Department of General Surgery, The First Affiliated Hospital of Anhui Medical University, Hefei, China; ^3^ Department of General Surgery, The Fourth Affiliated Hospital of Anhui Medical University, Hefei, China; ^4^ Department of Emergency Surgery, The Second Affiliated Hospital of Anhui Medical University, Hefei, China

**Keywords:** early gastric cancer (EGC), lymph node metastasis (LNM), risk factors, nomogram, premenopause

## Abstract

**Background:**

Lymph node metastasis (LNM) has a significant impact on the prognosis of patients with early gastric cancer (EGC). Our aim was to identify the independent risk factors for LNM and construct nomograms for male and female EGC patients, respectively.

**Methods:**

Clinicopathological data of 1,742 EGC patients who underwent radical gastrectomy and lymphadenectomy in the First Affiliated Hospital, Second Affiliated Hospital, and Fourth Affiliated Hospital of Anhui Medical University between November 2011 and April 2021 were collected and analyzed retrospectively. Male and female patients from the First Affiliated Hospital of Anhui Medical University were assigned to training sets and then from the Second and Fourth Affiliated Hospitals of Anhui Medical University were enrolled in validation sets. Based on independent risk factors for LNM in male and female EGC patients from the training sets, the nomograms were established respectively, which was also verified by internal validation from the training sets and external validation from the validation sets.

**Results:**

Tumor size (odd ratio (OR): 1.386, *p* = 0.030), depth of invasion (OR: 0.306, *p* = 0.001), Lauren type (OR: 2.816, *p* = 0.000), lymphovascular invasion (LVI) (OR: 0.160, *p* = 0.000), and menopause (OR: 0.296, *p* = 0.009) were independent risk factors for female EGC patients. For male EGC patients, tumor size (OR: 1.298, *p* = 0.007), depth of invasion (OR: 0.257, *p* = 0.000), tumor location (OR: 0.659, *p* = 0.002), WHO type (OR: 1.419, *p* = 0.001), Lauren type (OR: 3.099, *p* = 0.000), and LVI (OR: 0.131, *p* = 0.000) were independent risk factors. Moreover, nomograms were established to predict the risk of LNM for female and male EGC patients, respectively. The area under the ROC curve of nomograms for female and male training sets were 87.7% (95% confidence interval (CI): 0.8397–0.914) and 94.8% (95% CI: 0.9273–0.9695), respectively. For the validation set, they were 92.4% (95% CI: 0.7979–1) and 93.4% (95% CI: 0.8928–0.9755), respectively. Additionally, the calibration curves showed good agreements between the bias-corrected prediction and the ideal reference line for both training sets and validation sets in female and male EGC patients.

**Conclusions:**

Nomograms based on risk factors for LNM in male and female EGC patients may provide new insights into the selection of appropriate treatment methods.

## Introduction

Early gastric cancer (EGC) is classified as a gastric tumor confined to the mucosa or submucosa, regardless of lymph node metastasis (LNM). In recent years, endoscopic resection (ER), as an effective and safe minimally invasive approach, has been widely used in patients with EGC without LNM (1–3). Therefore, assessing the status of LNM is essential prior to ER or surgery. At present, computed tomography (CT), B-ultrasonography, enhanced CT, and endoscopic ultrasonography are the main examinations used to assess the clinical tumor-node-metastasis stage, including depth of invasion, LNM, and distant metastasis (4, 5). However, small metastatic lymph nodes or metastatic lymph nodes that have not increased in size cannot be accurately observed by these imaging methods. According to the recommendation of the Japanese Gastric Cancer Treatment Guidelines (6), the absolute indications for ER are as follows: differentiated adenocarcinoma, depth of invasion limited to the mucosa, tumor size of <2 cm, and without ulcers, thereby indicating an extremely low rate of LNM. A different study has also shown that the prognosis of patients with EGC can be affected by the incidence of LNM (7). Therefore, more factors need to be identified to evaluate LNM status.

Previous studies have explored the risk factors for LNM of EGC patients and established corresponding prediction models (8–11). Previous study reported that the female sex is an independent risk factor for LNM in patients with EGC (12). Besides, there is a difference in the incidence of EGC between male and female. For women, estrogen is higher during premenopause than during menopause (13). Estrogen has been shown to promote the development of GC (14). However, whether menopause is a new risk factor in GC remains unclear. In addition, it is necessary to establish a model for predicting the LNM of patients with EGC by gender. Due to its simple operation and intuitive image, nomogram is widely used to evaluate the prognosis of patients with a variety of diseases. In the present study, based on clinicopathologic data of 1,742 patients with EGC from three clinical centers, we established an effective nomogram prediction model for LNM in male and female EGC patients, respectively, assisting to choose a more precise treatment for EGC patients.

## Materials and Methods

### Patients

The clinical and pathological data of 16,281 GC patients who underwent radical gastrectomy and lymphadenectomy in three clinical centers (The First Affiliated Hospital, Second Affiliated Hospital, and Fourth Affiliated Hospital of Anhui Medical University, Hefei, China) between November 2011 and April 2021 were retrospectively collected. The exclusion criteria were as follows: (1) patients without complete clinical and pathological data; (2) patients with gastric stump carcinoma; (3) patients who had received neoadjuvant therapy; and (4) multiple primary tumors. Finally, a total of 1,742 patients with EGC were enrolled in the present study. Among them, 494 female and 1,248 male patients with EGC were identified. This study was approved by the Ethics Committee of The First Affiliated Hospital of Anhui Medical University.

### Clinicopathological Parameters

To determine the independent risk factors for LNM in EGC, the associations between different clinicopathological characteristics and LNM were analyzed. The following factors were examined in this study: age, sex, invasion depth, tumor size, tumor location, histological type, lymphovascular invasion (LVI), perineural invasion, LNM, ulcer, carcinoembryonic antigen (CEA), carbohydrate antigen 199 (CA199), carbohydrate antigen 125 (CA125), menopausal status, smoking, drinking alcohol, and family history of cancer. According to the World Health Organization classification for GC, the WHO types are polypoid, tubular, poorly differentiated, signet-ring cell, and mucinous adenocarcinoma (11). Besides, the Lauren type (intestinal, diffuse, and mixed type) was also included in this study. In addition, CEA, CA199, and CA125 were considered abnormal at over 5 ng/ml, 27 U/ml, and 35 U/ml, respectively.

### Statistical Analysis

Statistical analysis was performed using SPSS software (Version 22.0; IBM Corp.) and R software (Version 4.0.5). Measurement data are presented as the mean ± standard deviation. In univariate analysis, Pearson’s *χ* ^2^ or Fisher’s exact test was performed to analyze categorical variables, and the Students’ *t*-test or rank-sum test was used to examine continuous variables. Logistic regression was used for multivariate analysis to screen out the independent risk factors for LNM in EGC.

Furthermore, female and male patients with EGC from the First Affiliated Hospital of Anhui Medical University were assigned to the training sets, respectively. The independent risk factors in the training set were screened out by logistic regression. Based on the above independent risk factors, the nomogram prediction models were constructed to predict the risk of LNM in female and male patients with EGC, respectively. Additionally, the 246 patients from the Second Affiliated Hospital and Fourth Affiliated Hospital of Anhui Medical University were assigned to the test sets for external validation. The reliabilities of the nomogram prediction models were evaluated based on its discrimination and calibration. The concordance index, which is similar to the area under the receiver operating characteristic (ROC) curve, was calculated using the bootstrap resampling method to evaluate the discrimination ability of the model. Calibration curves were used to detect consistency between the actual LNM and the predicted LNM probability from the nomogram. The nomogram model was constructed using the “plotROC” package. The ROC curve was plotted using the “pROC” package, and the calibration curve was prepared using the “RMS” function package. *p* < 0.05 was considered statistically significant.

## Results

### Characteristics of 1,742 Patients With EGC From Three Clinical Centers

**Figure 1:**
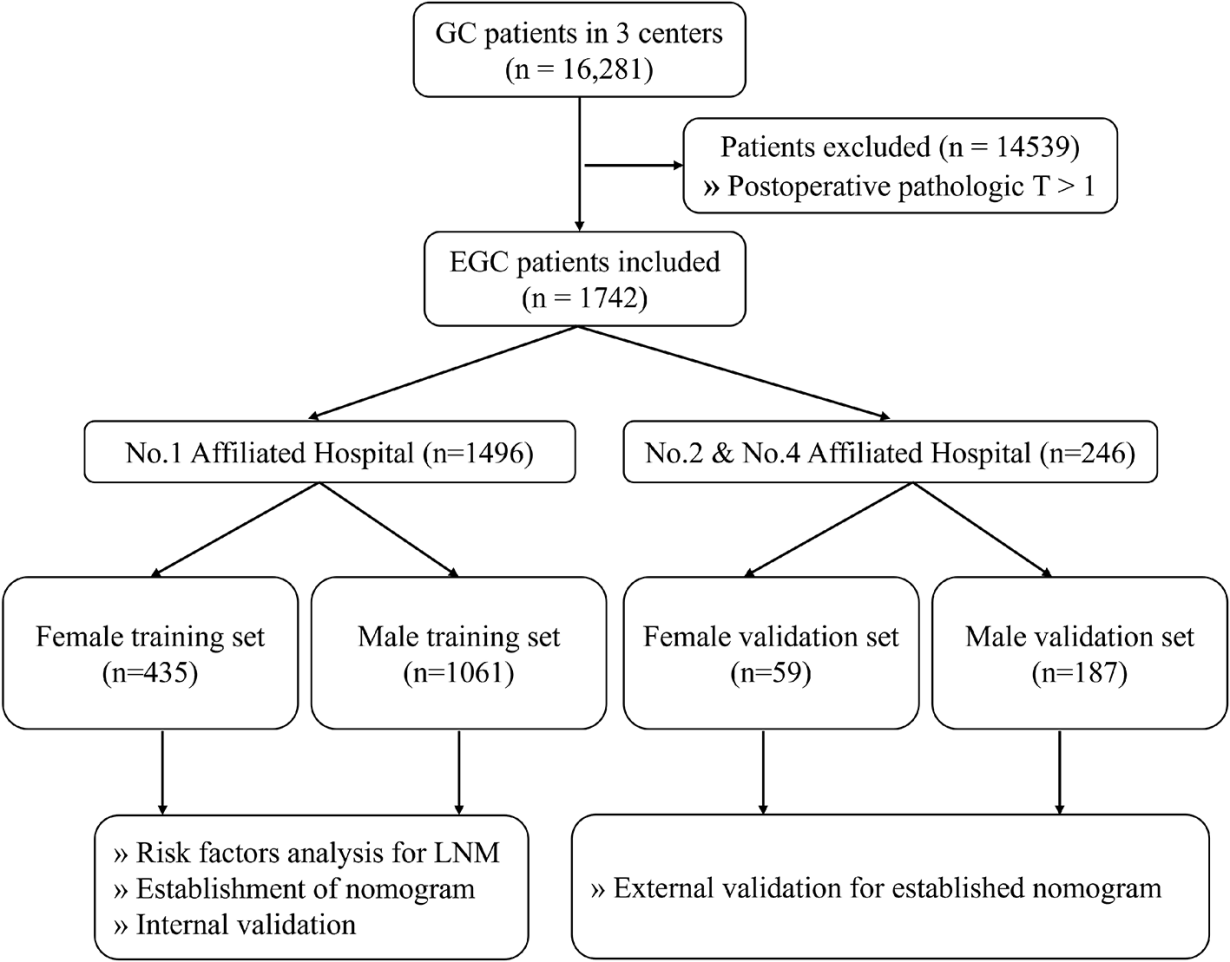
The flowchart of data collection and grouping for patients with EGC. EGC, early gastric cancer.

A total of 16,281 patients with GC were collected, among which 1,742 (10.7%) patients with EGC meeting the requirements were screened out. One thousand four hundred ninety-six (85.9%) of these patients enrolled into the training set were from the First Affiliated Hospital of Anhui Medical University and 246 (14.1%) of these patients enrolled into the validation set were from the Second and Fourth Affiliated Hospital of Anhui Medical University (**Figure 1**). Among the female patients, 435 (88.1%) were included in the training set and 59 (11.9%) were included in the validation set. Among the male patients, 1,061 (85.0%) were included in the training set and 187 (15.0%) were included in the validation set. Of the EGC patients, 12.3% (214/1742) were diagnosed with LNM totally, 10.4% (130/1248) for men and 17.0% (84/494) for women, respectively. The LNM rates of EGC patients in males and females were 10.2% (108/1061) and 17.7% (77/435) in the training sets and 11.8% (22/187) and 11.9% (7/59) in the validation sets, respectively (**Table 1**).

**Table 1:** Characteristic of 1,742 patients with EGC from three clinical centers.

Variables	No. 1 affiliated hospital (*n* = 1,496)		Nos. 2 and 4 affiliated hospital (*n* = 246)	
	LNM (−), *n* = 1,311	LNM (+), *n* = 185	LNM (−), *n* = 217	LNM (+), *n* = 29
Age (years old)	61.2 ± 11.2	58.6 ± 12.2	63.8 ± 10.1	59.4 ± 12.5
Tumor size (cm)	2.2 ± 1.1	2.7 ± 1.4	2.2 ± 1.3	2.7 ± 1.0
Gender				
Female	358 (27.3%)	77 (41.6%)	52 (24.0%)	7 (24.1%)
Male	953 (72.7%)	108 (58.4%)	165 (76.0%)	22 (75.9%)
Depth of invasion				
Mucosa	651 (49.7%)	37 (20.0%)	104 (47.9%)	6 (20.7%)
Submucosa	660 (50.3%)	148 (80.0%)	113 (52.1%)	29 (79.3%)
Ulceration				
No	662 (50.5%)	60 (32.4%)	141 (65.0%)	21 (72.4%)
Yes	649 (49.5%)	125 (67.6%)	76 (35.0%)	8 (27.6%)
Tumor location				
Upper	429 (32.7%)	23 (12.4%)	90 (41.5%)	10 (34.5%)
Middle	217 (16.6%)	27 (14.6%)	33 (15.2%)	5 (17.2%)
Lower	665 (50.7%)	135 (73.0%)	94 (43.3%)	14 (48.3%)
WHO type				
Polypoid adenocarcinoma	78 (5.9%)	3 (1.6%)	8 (3.7%)	1 (3.4%)
Tubular adenocarcinoma	870 (66.4%)	75 (40.5%)	154 (71.0%)	15 (51.7%)
Poorly differentiated	165 (12.6%)	44 (23.8%)	22 (10.1%)	5 (17.2)
Mucinous adenocarcinoma	106 (8.1%)	29 (15.7%)	14 (6.5%)	3 (10.3%)
Signet-ring cell carcinoma	92 (7.0%)	34 (18.4%)	19 (8.8%)	5 (17.2%)
Lauren type				
Intestinal	1,033 (78.8%)	18 (9.7%)	182 (83.9%)	4 (13.8%)
Diffuse	135 (10.3%)	132 (71.4%)	12 (5.5%)	19 (65.5%)
Mixed	143 (10.9%)	35 (9.7%)	23 (10.6%)	6 (20.7%)
LVI				
No	1,262 (96.3%)	132 (71.4%)	198 (91.2%)	15 (51.7%)
Yes	49 (3.7%)	53 (28.6%)	19 (8.8%)	14 (48.3%)
CEA				
<5 ng/ml	1,191 (90.8%)	163 (88.1%)	202 (93.1%)	23 (79.3%)
≥5 ng/ml	120 (9.2%)	22 (11.9%)	15 (6.9%)	6 (20.7%)
CA199				
<27 U/ml	1,256 (95.8%)	167 (90.3%)	210 (96.8%)	25 (86.2%)
≥27 U/ml	55 (4.2%)	18 (9.7%)	7 (3.2%)	4 (13.8%)
CA125				
<35 U/ml	1,292 (98.6%)	176 (95.1%)	214 (98.6%)	18 (62.1%)
≥35 U/ml	19 (1.4%)	9 (4.9%)	3 (1.4%)	11 (37.9%)
Family-tumor history				
No	1,266 (96.6%)	179 (96.8%)	211 (97.2%)	29 (100.0%)
Yes	45 (3.4%)	6 (3.2%)	6 (2.8%)	0 (0.0%)
Drinking				
No	1,060 (80.9%)	151 (81.6%)	189 (87.1%)	26 (89.7%)
Yes	251 (19.1%)	34 (18.4%)	28 (12.9%)	3 (10.3%)
Smoking				
No	1,009 (77.0%)	147 (79.5%)	187 (86.2%)	27 (73.1%)
Yes	302 (23.0%)	38 (20.5%)	30 (13.8%)	2 (6.9%)
Perineural invasion				
No	1,296 (98.9%)	176 (95.1%)	209 (96.3%)	26 (89.7%)
Yes	15 (1.1%)	9 (4.9%)	8 (3.7%)	3 (10.3%)

LNM, lymph node metastasis; EGC, early gastric cancer; LVI, lymphovascular invasion; CEA, carcinoembryonic antigen; CA199, carbohydrate antigen199; CA125, carbohydrate antigen 125.

### Construction and Validation of the Prediction Model for Female EGC Patients

In the training set of female EGC patients, univariate analysis suggested that age, tumor size, tumor location, WHO type, Lauren type, LVI, depth of invasion, presence of ulcers, and premenopause were associated with LNM (**Table 2**). Multivariate analysis indicated that tumor size (odd ratio (OR): 1.386, *p* = 0.030), depth of invasion (OR: 0.306, *p* = 0.001), Lauren type (OR: 2.816, *p* = 0.000), LVI (OR: 0.160, *p* = 0.000), and menopause (OR: 0.296, *p* = 0.009) were independent risk factors for female EGC patients (**Table 3**).

**Table 2:** Predictive variables for LNM in EGC patients of training set by gender.

Variables	Female EGC (*n* = 435)			Male EGC (*n* = 1,061)		
	LNM (−), *n* = 358	LNM (+), *n* = 77	*p*	LNM (−), *n* = 953	LNM (+), *n* = 108	*p*
Age	59.5 ± 13.0	54.8 ± 12.02	<0.001	61.9 ± 10.4	61.3 ± 11.6	0.619
Tumor size	2.06 ± 1.0	2.6 ± 1.3	0.004	2.2 ± 1.1	2.8 ± 1.5	<0.001
Depth of invasion			<0.001			<0.001
Mucosa	193 (53.9%)	20 (26.0%)		458 (48.1%)	17 (15.7%)	
Submucosa	165 (46.1%)	57 (74.0%)		495 (51.9%)	91 (84.3%)	
Ulceration			0.005			<0.001
No	184 (51.4%)	26 (33.8%)		478 (50.2%)	34 (31.5%)	
Yes	174 (48.6%)	51 (66.2%)		475 (49.8%)	74 (68.5%)	
Tumor location			0.001			<0.001
Upper	83 (23.2%)	5 (6.5%)		346 (36.3%)	18 (16.7%)	
Middle	75 (20.9%)	12 (15.6%)		142 (14.9%)	15 (13.9%)	
Lower	200 (55.9%)	60 (77.9%)		465 (48.8%)	75 (69.4%)	
WHO type			<0.001			<0.001
Polypoid adenocarcinoma	13 (3.6%)	1 (1.3%)		65 (6.8%)	2 (1.9%)	
Tubular adenocarcinoma	193 (53.9%)	20 (26.0%)		677 (71.0%)	55 (50.9%)	
Poorly differentiated	64 (17.9%)	23 (29.9%)		101 (10.6%)	21 (19.4%)	
Mucinous adenocarcinoma	45 (12.6%)	15 (19.5%)		61 (6.4%)	14 (13.0%)	
Signet-ring cell carcinoma	43 (12.0%)	18 (23.4%)		49 (5.1%)	16 (14.8%)	
Lauren type			<0.001			<0.001
Intestinal	259 (72.3%)	13 (16.9%)		774 (81.2%)	5 (4.6%)	
Diffuse	62 (17.3%)	49 (63.6%)		73 (7.7%)	83 (76.9%)	
Mixed	37 (10.3%)	15 (19.5%)		106 (11.1%)	20 (18.5%)	
LVI			<0.001			<0.001
No	342 (95.5%)	53 (68.8%)		920 (96.5%)	79 (73.1%)	
Yes	16 (4.5%)	24 (31.2%)		33 (3.5%)	29 (26.9%)	
CEA			0.335			0.211
<5 ng/ml	340 (95.0%)	71 (92.2%)		851 (89.3%)	92 (85.2%)	
≥5 ng/ml	18 (5.0%)	6 (7.8%)		102 (10.7%)	16 (14.8%)	
CA199			0.028			0.031
<27 U/ml	340 (95.0%)	68 (88.3%)		916 (96.1%)	99 (91.7%)	
≥27 U/ml	18 (5.0%)	9 (11.7%)		37 (3.9%)	9 (8.3%)	
CA125			0.051			0.027
<35 U/ml	354 (98.9%)	73 (94.8%)		938 (98.4%)	103 (95.4%)	
≥35 U/ml	4 (1.1%)	4 (5.2%)		15 (1.6%)	5 (4.6%)	
Family-tumor history			0.559			0.735
No	335 (93.6%)	74 (96.1%)		931 (97.7%)	105 (97.2%)	
Yes	23 (6.4%)	3 (3.9%)		22 (2.3%)	3 (2.8%)	
Drinking			0.591			0.205
No	353 (98.6%)	77 (100.0%)		707 (74.2%)	74 (68.5%)	
Yes	5 (1.4%)	0 (0%)		246 (25.8%)	34 (31.5%)	
Smoking			0.297			0.695
No	356 (99.4%)	75 (97.4%)		653 (68.5%)	72 (66.7%)	
Yes	2 (0.6%)	2 (2.6%)		300 (31.5%)	36 (33.3%)	
Perineural invasion			0.051			0.005
No	354 (98.9%)	73 (94.8%)		942 (98.8%)	103 (95.4%)	
Yes	4 (1.1%)	4 (5.2%)		11 (1.2%)	5 (4.6%)	
Menopause			<0.001			
Premenopause	77 (21.5%)	41 (53.2%)		–	–	
Postmenopause	281 (78.5%)	36 (46.8%)		–	–	

LMN, lymph node metastasis; EGC, early gastric cancer; LVI, lymphovascular invasion; CEA, carcinoembryonic antigen; CA199, carbohydrate antigen199; CA125, carbohydrate antigen 125. Red font text means statistically significant.

**Table 3:** Multivariate analysis for LNM in female training set with EGC.

Variables	*B*	SE	Wald	*df*	Sig.	Exp (*B*)
Age	−0.005	0.018	0.066	1	0.797	0.995
Tumor size	0.326	0.150	4.736	1	0.030	1.386
Depth of invasion (submucosa)	−1.183	0.370	10.207	1	0.001	0.306
Ulceration (positive)	−0.029	0.347	0.007	1	0.932	0.971
Tumor location (lower)	−0.481	0.266	3.256	1	0.071	0.618
WHO type	0.185	0.137	1.810	1	0.179	1.203
Lauren type (mixed and diffuse)	1.035	0.209	24.495	1	0.000	2.816
LVI (positive)	−1.832	0.479	14.638	1	0.000	0.160
CA199 (over 27 U/ml)	0.659	0.575	1.313	1	0.252	1.932
CA125 (over 35 U/ml)	0.957	0.965	0.982	1	0.322	2.603
Perineural invasion (positive)	0.578	1.048	0.304	1	0.581	1.783
Menopause (premenopause)	−1.217	0.463	6.918	1	0.009	0.296

Red font text means statistically significant.

**Figure 2:**
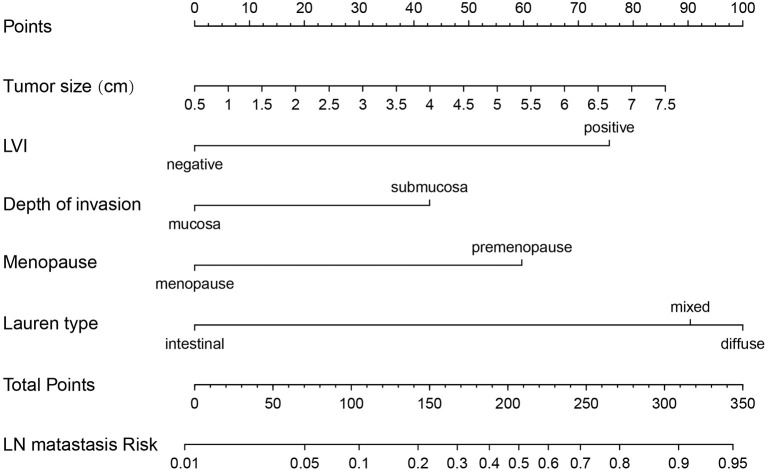
Nomogram prediction model for LN metastasis in female EGC patients. LN, lymph node; LVI, lymphovascular invasion; EGC, early gastric cancer.

**Figure 3:**
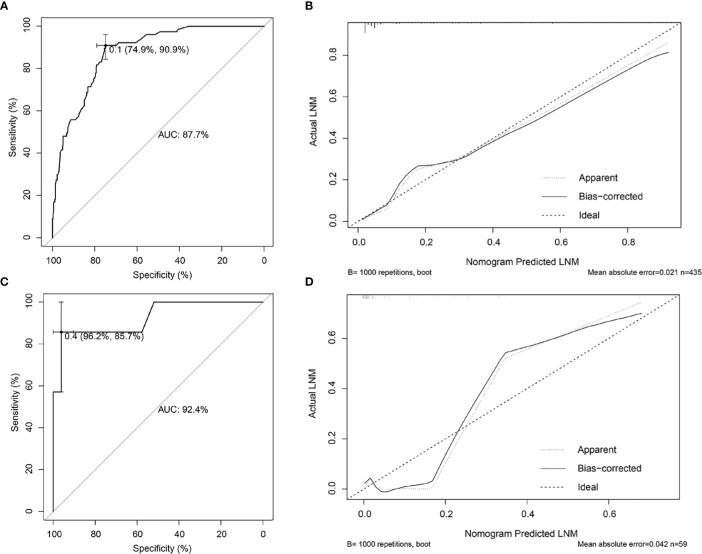
Internal and external validations for the nomogram prediction model in female EGC patients. **(A)** ROC curve of the nomogram prediction model in the training set of female EGC patients; the AUC was 87.7% (95% CI: 0.8397–0.914). **(B)** Calibration curve of the nomogram prediction model for the training set of female EGC patients. **(C)** ROC curve of the nomogram prediction model for female EGC patients from the validation set; the AUC was 92.4% (95% CI: 0.7979–1). **(D)** Calibration curve of the nomogram prediction model in the validation set of female EGC patients. LNM, lymph node metastasis; EGC, early gastric cancer; ROC, receiver operating characteristic; AUC, area under the ROC curve.

Risk factors screened out by multivariate analysis were used to construct nomogram prediction model for LNM in female EGC patients. In nomogram, the first line (points) ranged from 0 to 100, providing corresponding points for the variables of the following lines. Patients’ points in each variable were added up to the total points. The scale aligned with the total points on the risk line is the predicted risk of EGC patients occurring LNM. In the nomogram, total points ranged from 0 to 350 for female EGC patients. A female patient with a tumor reaching 2 cm got 17.5 points, and the risk for LNM was about 5%. Among the categorical variables, the effect of Lauren type (mixed and diffuse type) on female patients was the most significant factor (**Figure 2**). For internal validation from the training set and external validation from the validation set, the area under the ROC curve was 87.7% (95% confidence interval (CI): 0.8397–0.914) and 92.4% (95% CI: 0.7979–1), respectively (**Figures 3A, C**). The calibration curve which compared the predicted probability of LNM with the actual probability, showed good agreements between the bias-corrected prediction and the ideal reference line for both training set and validation set (**Figures 3B, D**).

### Construction and Validation of the Prediction Model for LNM in Male EGC Patients

As for the training set, univariate analysis showed that tumor size, tumor location, LVI, depth of invasion, histological types, presence of ulcers, WHO type, Lauren type, CA199, CA125, and perineural invasion had an association with LNM (**Table 2**). Multivariate analysis demonstrated that tumor size (OR: 1.298, *p* = 0.007), depth of invasion (OR: 0.257, *p* = 0.000), tumor location (OR: 0.659, *p* = 0.002), WHO type (OR: 1.419, *p* = 0.001), Lauren type (OR: 3.099, *p* = 0.000), and LVI (OR: 0.131, *p* = 0.000) were independent risk factors for male EGC patients (**Table 4**). Tumor size, Lauren type, LVI, and invasion depth were independent risk factors for both male and female EGC patients.

**Table 4:** Multivariate analysis for LNM in male training set with EGC.

Variables	*B*	SE	Wald	*df*	Sig.	Exp (*B*)
Tumor size	0.261	0.096	7.394	1	0.007	1.298
Depth of invasion (submucosa)	−1.359	0.302	20.282	1	0.000	0.257
Tumor location (lower)	−0.417	0.154	7.323	1	0.002	0.659
WHO type	0.35	0.112	9.77	1	0.001	1.419
Lauren type (mixed and diffuse)	1.131	0.146	60.335	1	0.000	3.099
LVI (positive)	−2.035	0.367	30.742	1	0.000	0.131
CA125 (over 35 U/ml)	0.001	0.006	0.023	1	0.879	1.001
CA199 (over 27 U/ml)	0.000	0.003	0.012	1	0.914	1.000
Perineural invasion (positive)	0.270	0.716	0.142	1	0.707	1.310

Red font text means statistically significant.

**Figure 4:**
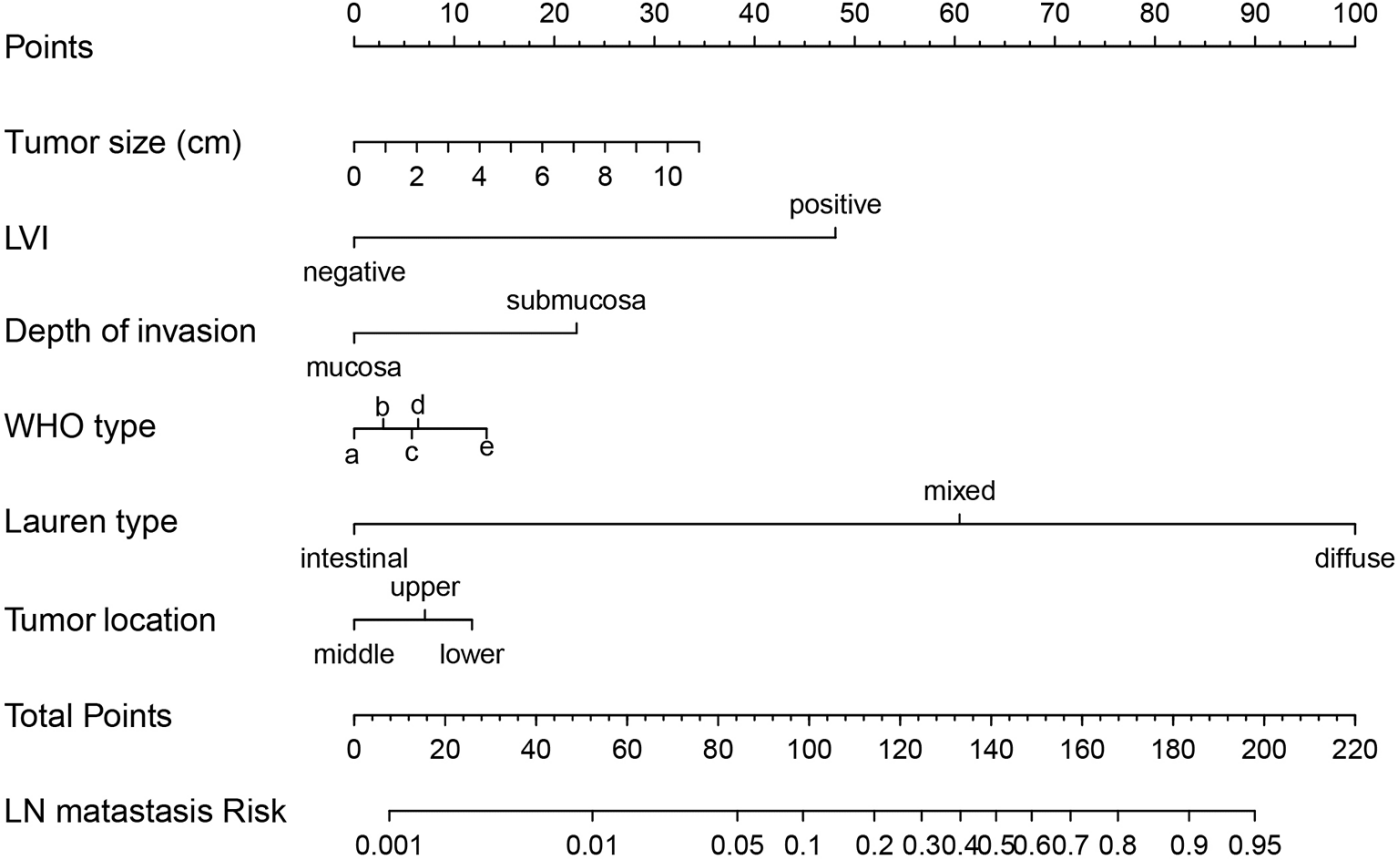
Nomogram prediction model for LN metastasis in male EGC patients. LN, lymph node; EGC, early gastric cancer; LVI, lymphovascular invasion; WHO types: (a) polypoid adenocarcinoma; (b) tubular adenocarcinoma; (c) poorly differentiated; (d) mucinous adenocarcinoma; (e) signet-ring cell carcinoma.

**Figure 5:**
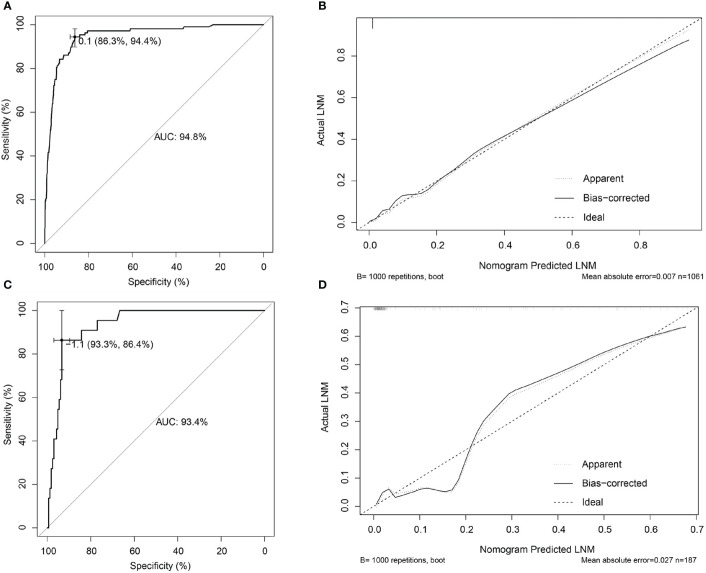
Internal and external validations for the nomogram prediction model in male EGC patients. **(A)** ROC curve of the nomogram prediction model in the training set of male EGC patients; the AUC was 94.8% (95% CI: 0.9273–0.9695). **(B)** Calibration curve of the nomogram prediction model for the training set of male EGC patients. **(C)** ROC curve of the nomogram prediction model for male EGC patients in the validation set; the AUC was 93.4% (95% CI: 0.8928–0.9755). **(D)** Calibration curve of the nomogram prediction model in the validation set of male EGC patients. LNM, lymph node metastasis; EGC, early gastric cancer; ROC, receiver operating characteristic; AUC, area under the ROC curve.

Independent risk factors were incorporated into the construction of nomogram to obtain a risk prediction model for male EGC patients. In the nomogram, total points ranged from 0 to 220 for male EGC patients. When the LVI was positive, male patients with EGC scored 100 points (**Figure 4**). Among the categorical variables, Lauren type (diffuse) scores the highest. The area under the ROC curve was 94.8% (95% CI: 0.9273–0.9695) for the training set and 93.4% (95% CI: 0.8928–0.9755) for the validation set, respectively (**Figures 5A, C**). The calibration curve showed good agreements between the bias-corrected prediction and the ideal reference line for both training set and validation set (**Figures 5B, D**).

## Discussion

Currently, GC is the fifth most common type of cancer and the fourth most common cause of cancer-related mortality worldwide (15). With the advancements in the diagnosis and treatment of GC, GC morbidity and mortality have declined in recent years (15–17). In Japan, the early diagnosis rate of GC is >50%; by contrast, the same rate in China is only ~10%, which may lead to a poor 5-year survival rate (18, 19). More screening programs may help improve the diagnostic rate of EGC and lead to an improved prognosis, which may also influence the results of studies further exploring independent risk factors for LNM in EGC.

In addition to gastrectomy, ER is the main treatment method to treat EGC and is appropriate for EGC with a low LNM rate, including endoscopic submucosal dissection (ESD) and endoscopic mucosal resection (EMR). According to the Japanese Gastric Cancer Treatment Guidelines 2018 (5th edition) (6), the absolute indications for ESD and EMR are a differentiated-type EGC with an infiltration level limited to the mucosa, a tumor size of ≤2 cm, and no presence of ulcers. Absolute indications of ESD also include a differentiated-type mucosal EGC without the prevalence of ulcers with a tumor size of >2 cm, and a differentiated-type mucosal EGC with a prevalence of ulcers and a tumor size of ≤2 cm. Compared with gastrectomy, EMR and ESD are more minimally invasive, significantly improving EGC patients’ quality of life (20, 21). EMR and ESD have been widely used in recent years with the gradual indication expansion. However, the use of ER in patients with expanded indications is controversial, due to the lack of long-term evidence of its safety (20–23). In the present study, 512 patients met the absolute indications and 15 (2.9%) had LNM, whose possibility was higher than the 1% possibility required for absolute indications (6). Compared with Japan, the diagnostic rate of EGC in China is relatively low, resulting in a relatively low sample size. In addition, different from the trials in Japan using ER (24), all patients in this study underwent radical gastrectomy, and the differences in the corresponding inclusion criteria may also lead to differences between the results.

The incidence of male GC is known to be higher than that of female GC, but the mortality rate of female patients with GC is higher than that of male patients (15–17). In this study, female patients with EGC had a higher LNM ratio than male patients (17.0% vs. 10.4%), which was consistent with previous studies (8, 10, 25). It is therefore necessary to analyze the risk factors for LNM in male and female patients with EGC separately. Menopausal status is a critical characteristic in female compared with male patients. However, few studies have reported the effect of menopausal status on LNM in EGC. In the present study, it was found that the LNM ratio of premenopausal female patients (32.6%, 47/144) was higher than that of male (10.4%, 130/1248) and postmenopausal female patients (10.6%, 37/350). Age is associated with menopausal status, indicating younger age might complicate the relationship between menopausal status and LNM. Therefore, through multivariate analysis, it was identified that premenopausal status, not age, was an independent risk factor for LNM in female patients with EGC. Zhang et al. demonstrated that estrogen can stimulate the secretion of IL-6 from GC-associated fibroblasts, and then activate the STAT3 signaling pathway, resulting in enhanced GC cell proliferation and invasion (26). Furthermore, the expression of estrogen receptor-α36 has been reported to be highly correlated with LNM in GC (14), which may be helpful for predicting the risk of LNM in GC in the future. Further studies on the mechanism of estrogen and its receptors will provide new insights for the treatment of GC. Due to the limitations of retrospective studies on data collection, it was regrettable that sex hormone levels and use of oral contraceptives cannot be analyzed in this study.

In the male population with EGC, tumor size, depth of invasion, tumor location, WHO type, Lauren type, and LVI were independent risk factors for LNM. Among the WHO type and Lauren type, signet-ring cell carcinoma and diffuse type owned the greatest risk of LNM, respectively (**Figure 4**) due to their high lymph tropism and infiltrating behavior. Therefore, the extension of gastric resection might be more beneficial for EGC patients with diffuse type and signet-ring cell carcinoma (27). LVI, as another contraindication for ER, is easily ignored before surgery. In the EGC patients with LVI, the risk for LNM reached >10% and >70% in male and female EGC patients, respectively (**Figures 2** and **4**), which was similar with the results from Ren et al. (28), and LVI might be considered an evaluation index for effective removal of EGC after ER. When LVI is positive, the extension of gastric resection and lymph node dissection are necessary.

## Conclusions

In the present study, we analyzed the independent risk factors for LNM in female and male EGC patients, respectively. Importantly, menopausal status was firstly identified as an independent risk factor for LNM in female population with EGC. Additionally, based on the above risk factors, the nomograms were established for predicting risk of LNM in female and male EGC patients, which might be beneficial for selecting a more precise treatment.

## Data Availability Statement

The original contributions presented in the study are included in the article/supplementary material. Further inquiries can be directed to the corresponding author.

## Ethics Statement

The studies involving human participants were reviewed and approved by the Ethics Committee of The First Affiliated Hospital of Anhui Medical University. The patients/participants provided their written informed consent to participate in this study.

## Author Contributions

WH and ZC designed this study and revised this manuscript. WS, ZC, and CL collected and analyzed the data and drafted and revised this manuscript. PC, KS, ZW, HL, and JH were engaged in the collection of the clinical data. All authors contributed to the article and approved the submitted version.

## Funding

This work was supported by the Natural Science Project of Anhui Province (No. KJ2017A829).

## Conflict of Interest

The authors declare that the research was conducted in the absence of any commercial or financial relationships that could be construed as a potential conflict of interest.

## Publisher’s Note

All claims expressed in this article are solely those of the authors and do not necessarily represent those of their affiliated organizations, or those of the publisher, the editors and the reviewers. Any product that may be evaluated in this article, or claim that may be made by its manufacturer, is not guaranteed or endorsed by the publisher.

## References

[1] Meng FS Zhang ZH Wang YM Lu L Zhu J Ji F. Comparison of Endoscopic Resection and Gastrectomy for the Treatment of Early Gastric Cancer: A Meta-Analysis. Surg Endosc (2016) 30(9):3673–83. doi: 10.1007/s00464-015-4681-0 26659235

[2] Gotoda T Yanagisawa A Sasako M Ono H Nakanishi Y Shimoda T. Incidence of Lymph Node Metastasis From Early Gastric Cancer: Estimation With a Large Number of Cases at Two Large Centers. Gastric Cancer (2000) 3(4):219–25. doi: 10.1007/PL00011720 11984739

[3] Zhao BW Chen YM Jiang SS Chen YB Zhou ZW Li YF. Lymph Node Metastasis, a Unique Independent Prognostic Factor in Early Gastric Cancer. PLoS One (2015) 10(7):e0129531. doi: 10.1371/journal.pone.0129531 26154617 PMC4496056

[4] Kim AY Kim HJ Ha HK. Gastric Cancer by Multidetector Row CT: Preoperative Staging. Abdom Imaging (2005) 30(4):465–72. doi: 10.1007/s00261-004-0273-5 15785907

[5] Cardoso R Coburn N Seevaratnam R Sutradhar R Lourenco LG Mahar A. A Systematic Review and Meta-Analysis of the Utility of EUS for Preoperative Staging for Gastric Cancer. Gastric Cancer (2012) 15(Suppl 1):S19–26. doi: 10.1007/s10120-011-0115-4 22237654

[6] Japanese Gastric Cancer A. Japanese Gastric Cancer Treatment Guidelines 2018 (5th Edition). Gastric Cancer (2021) 24(1):1–21. doi: 10.1007/s10120-020-01042-y 32060757 PMC7790804

[7] Li X Liu S Yan J Peng L Chen M Yang J. The Characteristics, Prognosis, and Risk Factors of Lymph Node Metastasis in Early Gastric Cancer. Gastroenterol Res Pract (2018) 2018:6945743. doi: 10.1155/2018/6945743 29853864 PMC5954923

[8] Chen J Zhao G Wang Y. Analysis of Lymph Node Metastasis in Early Gastric Cancer: A Single Institutional Experience From China. World J Surg Oncol (2020) 18(1):57. doi: 10.1186/s12957-020-01834-7 32197625 PMC7085136

[9] Kim SM Lee H Min BH Kim JJ An JY Choi MG. A Prediction Model for Lymph Node Metastasis in Early-Stage Gastric Cancer: Toward Tailored Lymphadenectomy. J Surg Oncol (2019) 120(4):670–5. doi: 10.1002/jso.25628 31301150

[10] Gu L Chen M Khadaroo PA Ma X Kong L Li X. A Risk-Scoring Model for Predicting Lymph Node Metastasis in Early Gastric Cancer Patients: A Retrospective Study and External Validation. J Gastrointest Surg (2018) 22(9):1508–15. doi: 10.1007/s11605-018-3816-8 29845571

[11] Lin JX Wang ZK Wang W Desiderio J Xie J Wang J. Risk Factors of Lymph Node Metastasis or Lymphovascular Invasion for Early Gastric Cancer: A Practical and Effective Predictive Model Based on International Multicenter Data. BMC Cancer (2019) 19(1):1048. doi: 10.1186/s12885-019-6147-6 31694573 PMC6836519

[12] Han WX Xu A Chen ZM Wei ZJ Liu H. Stratifying Risk and Establishing Predictive Risk-Scoring Model for Lymph-Node Metastasisin Early Gastric Cancer. Chin J Gen Surg (2017) 32):285–8. doi: 10.3760/cma.j.issn.1007-631X.2017.04.001

[13] Lephart ED. A Review of the Role of Estrogen in Dermal Aging and Facial Attractiveness in Women. J Cosmet Dermatol (2018) 17(3):282–8. doi: 10.1111/jocd.12508 29436770

[14] Deng H Huang X Fan J Wang L Xia Q Yang X. A Variant of Estrogen Receptor-Alpha, ER-Alpha36 Is Expressed in Human Gastric Cancer and Is Highly Correlated With Lymph Node Metastasis. Oncol Rep (2010) 24(1):171–6. doi: 10.3892/or_00000842 PMC3380086 20514458

[15] Sung H Ferlay J Siegel RL Laversanne M Soerjomataram I Jemal A. Global Cancer Statistics 2020: GLOBOCAN Estimates of Incidence and Mortality Worldwide for 36 Cancers in 185 Countries. CA Cancer J Clin (2021) 71:209–49. doi: 10.3322/caac.21660 33538338

[16] Bray F Ferlay J Soerjomataram I Siegel RL Torre LA Jemal A. Global Cancer Statistics 2018: GLOBOCAN Estimates of Incidence and Mortality Worldwide for 36 Cancers in 185 Countries. CA Cancer J Clin (2018) 68(6):394–424. doi: 10.3322/caac.21492 30207593

[17] Chen W Zheng R Baade PD Zhang S Zeng H Bray F. Cancer Statistics in China, 2015. CA Cancer J Clin (2016) 66(2):115–32. doi: 10.3322/caac.21338 26808342

[18] Luo M Li L. Clinical Utility of Miniprobe Endoscopic Ultrasonography for Prediction of Invasion Depth of Early Gastric Cancer: A Meta-Analysis of Diagnostic Test From PRISMA Guideline. Med (Baltimore) (2019) 98(6):e14430. doi: 10.1097/MD.0000000000014430 PMC6380697 30732202

[19] Zong L Abe M Seto Y Ji J. The Challenge of Screening for Early Gastric Cancer in China. Lancet (2016) 388(10060):2606. doi: 10.1016/S0140-6736(16)32226-7 27894662

[20] Takizawa K Ono H Muto M. Current Indications of Endoscopic Submucosal Dissection for Early Gastric Cancer in Japan. Jpn J Clin Oncol (2019) 49(9):797–802. doi: 10.1093/jjco/hyz100 31322655

[21] Choi JH Kim ES Lee YJ Cho KB Park KS Jang BK. Comparison of Quality of Life and Worry of Cancer Recurrence Between Endoscopic and Surgical Treatment for Early Gastric Cancer. Gastrointest Endosc (2015) 82(2):299–307. doi: 10.1016/j.gie.2015.01.019 25892060

[22] Nishizawa T Yahagi N. Long-Term Outcomes of Using Endoscopic Submucosal Dissection to Treat Early Gastric Cancer. Gut Liver (2018) 12(2):119–24. doi: 10.5009/gnl17095 PMC5832335 28673068

[23] Hahn KY Park CH Lee YK Chung H Park JC Shin SK. Comparative Study Between Endoscopic Submucosal Dissection and Surgery in Patients With Early Gastric Cancer. Surg Endosc (2018) 32(1):73–86. doi: 10.1007/s00464-017-5640-8 28639042

[24] Takizawa K Takashima A Kimura A Mizusawa J Hasuike N Ono H. A Phase II Clinical Trial of Endoscopic Submucosal Dissection for Early Gastric Cancer of Undifferentiated Type: Japan Clinical Oncology Group Study JCOG1009/1010. Jpn J Clin Oncol (2013) 43(1):87–91. doi: 10.1093/jjco/hys189 23166384

[25] Ryu ES Chang SJ An J Yang JY Chung JW Kim YJ. Sex-Specific Differences in Risk Factors of Lymph Node Metastasis in Patients With Early Gastric Cancer. PLoS One (2019) 14(10):e0224019. doi: 10.1371/journal.pone.0224019 31626653 PMC6799917

[26] Zhang Y Cong X Li Z Xue Y. Estrogen Facilitates Gastric Cancer Cell Proliferation and Invasion Through Promoting the Secretion of Interleukin-6 by Cancer-Associated Fibroblasts. Int Immunopharmacol (2020) 78:105937. doi: 10.1016/j.intimp.2019.105937 31753587

[27] Mengardo V Treppiedi E Bencivenga M Dal Cero M Giacopuzzi S. Tailored Treatment for Signet Ring Cell Gastric Cancer. Updates Surg (2018) 70(2):167–71. doi: 10.1007/s13304-018-0550-4 29948660

[28] Ren MH Qi XS Chu YN Yu Y Chen Y Zhang P. Risk of Lymph Node Metastasis and Feasibility of Endoscopic Treatment in Ulcerative Early Gastric Cancer. Ann Surg Oncol (2021) 28(4):2407–17. doi: 10.1245/s10434-020-09153-7 PMC7940277 32975685

